# Assessment of hydrological, geological, and biological parameters of a river basin impacted by old Hg mining in NW Spain

**DOI:** 10.1007/s11356-024-31888-z

**Published:** 2024-01-13

**Authors:** Lucía Escudero, Alfredo F. Ojanguren, Rodrigo Álvarez, Carmen García, Jose Pañeda, Fernando Alberquilla, Almudena Ordóñez

**Affiliations:** 1 Department Exploitation and Prospecting of Mines, University of Oviedo, Asturias, Spain; 2 Department Biology of Organisms and Systems, University of Oviedo, Asturias, Spain

**Keywords:** Abandoned metal mining, Fluvial sediments, Arsenic, Mercury, Ecological state, Environmental risk

## Abstract

Mercury (Hg) is a toxic metal that can cause adverse effects for the health of ecosystems. The Caudal River is one of the main rivers in the Asturias region (NW Spain), whose basin is highly anthropized, hosting several Hg mines, closed in the last century. Arsenic (As) is also found in the mineral paragenesis of the Hg deposits, posing a greater environmental risk. In the mining sites, remaining old facilities and tailings continue to release these elements into the environment. In this work, samples of fluvial sediments and water were taken, both in areas affected by anthropic activity and in pristine areas, in order to establish the background levels for the critical elements. The mineralogical study of the sediments, combined with EDS microanalysis, is useful to identify mineralogical traps such as Fe oxides or clays to retain the As. The As content in all sampled sediments is above the threshold effect levels (TEL), the possible effect range within which adverse effects occasionally occur, according to the Canadian Sediment Quality Guidelines for the Protection of Aquatic Life. This fact is related to a naturally high geochemical background in the basin, due to the existing mineral deposits. The PEL threshold (the probable effect range within which adverse effects frequently occur) is exceeded by more than an order of magnitude in the sediments downstream of the Hg mines. In these points, the As content in the water, exceeding 700 μg L^−1^ As, is also above the quality standard established in Spanish legislation. As a result, the Caudal River tributaries in the lower part of the basin do not reach a good ecological state, according to the Hydrological Planning Office, and in some cases their state is deficient, showing low richness and high dominance of macroinvertebrates. Although the concentrations decrease with distance from the source, these findings justify the low ecological quality of the affected watercourses.

**Supplementary Information:**

The online version contains supplementary material available at 10.1007/s11356-024-31888-z.

## Introduction 

**Fig. 1:**
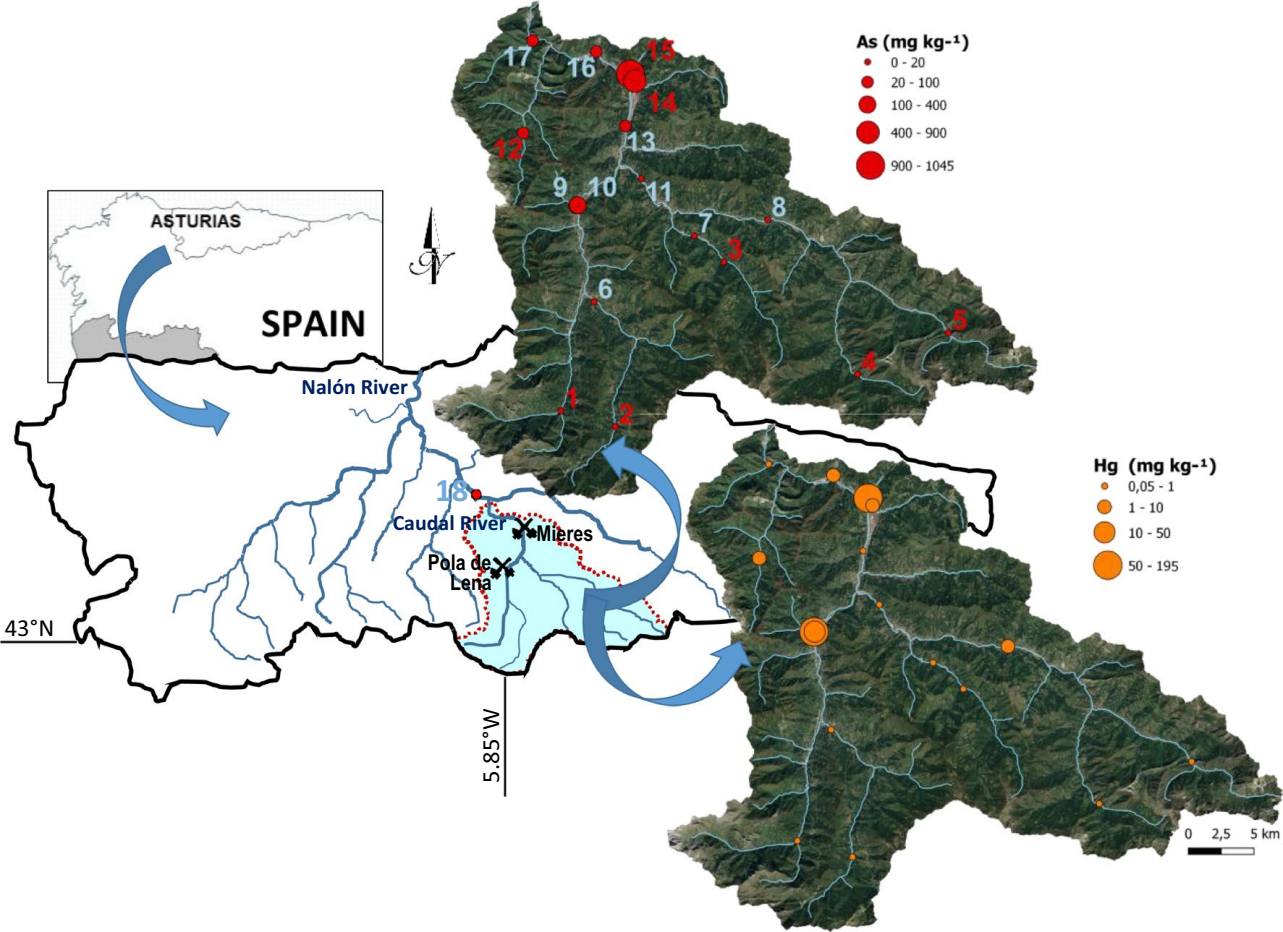
Location of the area of study. Caudal River basin (light blue shaded area). Representation of the As and Hg concentrations in the sediment samples (nos. 1 to 17). The water samples are represented by blue numbers

Mining is one of the main sources of metal input into the environment (Odumo et al. 2014; Rodríguez-Martín et al. 2013a). Wastes containing metals and metalloids found in abandoned mining sites constitute a persistent origin of pollution, posing a significant threat to both human well-being and the ecosystem (Campos-Herrera et al. 2016). Asturias (NW Spain) is a traditional mining region, with many closed coal and metal mines. Due to the abundance of Fe and coal resources, there was also metal-processing industries in the Caudal River basin, near the village of Mieres. Metal mining has not been as important economically as coal mining, but it has been relevant from an environmental point of view, especially Hg mining. Asturias was the second Hg producer of Spain and the third of the world in the 1960s (Loredo et al. 2003), being the most productive mines located in Mieres and Lena districts, within the Caudal River basin (Fig. 1), where there was also metallurgical activity. In the mineral paragenesis of these Hg deposits, As species are also found. All Asturian Hg mines were closed in the early 1970s, but the rest of the metallurgical installations and spoil heaps remain on site in most cases (Ordóñez et al. 2013). Intermittent mining of Cu–Co–Ni and, to a lesser extent, Sb, took place in the Caudal River basin. However, it was Hg mining that drove metal mining in this area. Therefore, this paper focuses mainly on Hg and As, which are highly toxic pollutants that can cause adverse health effects (Yu et al. 2022; Raj and Maiti 2019). Long-term kidney damage and neurological disorders have been observed following oral and dermal exposure to inorganic Hg (Garcia-Bravo et al. 2010; WHO 2003), whereas prolonged exposure by ingestion of As is associated with an increased risk of skin, lung, bladder, and kidney cancer (WHO 2001). The abundance of these elements in the continental crust of the earth is about 0.02–0.06 and 1–2 mg kg^−1^, respectively (Kabata-Pendias and Mukherjee 2007; Yaroshevsky 2006; National Research Council (USA) Committee on Medical and Biological Effects of Environmental Pollutants 1977; Masuda 2018). They are naturally scarce, but anthropogenic activities, such as mining, play an important role in dispersing these elements to the environment, including the hydrosphere.

Some trace elements such as Hg or As are not essential for the metabolism of organisms in aquatic ecosystems and are not under homeostatic control, so they are considered pollutants due to their persistence, toxicity, and bioconcentration, which is cause for global concern (Juncos et al. 2023). In particular, As contamination of aquatic systems is considered a major environmental problem affecting more than 115 countries worldwide (Hussain et al. 2021; Zhang et al. 2022), since it is highly soluble in aquatic environments, so it is easily released into natural waters (Masuda 2018). Abandoned metal mines represent a noteworthy origin of As pollution, since precipitation and wind transport it from the mining locations to the neighboring areas, posing health and ecological risks (Hoang et al. 2021). In this case, the instability of As-rich sulfide minerals remaining in the spoil heaps, together with the abundant rainfall in Asturias, results in the generation of As-rich leachates which are finally incorporated into the Caudal River. In contrast, cinnabar (Hg sulfide), which is the main ore of this metal, is highly insoluble, soHg mobility is low compared to that of As. Notwithstanding, cinnabar particles, being quite resistant to weathering, are often observed in the stream sediments, so high Hg concentrations can be found in the sediments downstream of mine sites (USGS 2016). In addition, the calcination of cinnabar ore often generates huge amounts of mining waste containing large amounts of fine-grained secondary Hg phases (soluble Hg salts) that also constitute sources of Hg to the local environment (Li et al. 2012). Hg or As settled to the sediment might be absorbed by phytoplankton or ingested by zooplankton or fish.

This work aims to holistically study the hydrological, geological, and biological aspects of a naturally mineralized and highly anthropized river basin. The main objectives of this study are i) to know the presence and distribution of toxic elements in river waters and sediments throughout the basin, based on sampling and analysis; ii) to identify both natural and anthropogenic sources of these elements; iii) to relate the above information to biodiversity through the study of macroinvertebrates in the basin; and iv) to determine, based on the information obtained, whether there is environmental risk to aquatic ecosystems.

## Materials and methods

### Area of study

The climate of this area is temperate (average temperature around 13 °C) and wet, characterized by abundant rainfall (about 1000 mm per year), so the rivers are perennial and of abundant flow. The Caudal River is a tributary of the Nalón River, the main watercourse of the region. The area drained by the Caudal River is a basin of 654 km^2^, which covers populated and industrialized sectors and those affected by Hg, Cu, and coal mining. The average flow of this river is around 20 m^3^ s^−1^.

Concerning the geological context, in the Caudal River basin there are calcareous outcrops on the southern and eastern limits, close to the divide, in the highest areas. Most of the subsoil of the basin is constituted by detrital sedimentary rocks, mostly of coastal and marine environments, from the Carboniferous system (sandstones, graywackes, and shales, essentially). At the mouth of the Caudal River, a wide calcareous outcrop is crossed again in the Aramo Mountain Range. In the Hg deposits, exploited since Roman times, this metal occurs mainly in the form of cinnabar (sulfide), although native Hg has also been occasionally found. In addition, it is worth noting the existence of significant amounts of As, mainly in the form of realgar, orpiment, arsenopyrite, and As-rich pyrite. Other sulfides, such as galena, chalcopyrite, and sphalerite are also found in the paragenesis of these Hg deposits (Loredo et al. 2003).

Regarding the hydrogeology, there are no aquifers of importance in this area. The predominance of low permeability rocks of Carboniferous age means that surface runoff predominates over infiltration (Ordóñez et al. 2013), so the surface mobility of Hg and As will be greater than that of the groundwater.

A total of 14 fluvial sediment samples and 10 water samples were taken in the Caudal River catchment (Fig. 1; the UTM coordinates of the sampling points are available as Supplementary Material). Five of the sediment samples (numbers 1 to 5) were taken at the head of the basin, in areas not anthropically affected, in order to define background values. Samples 9 and 10 were taken right downstream of the main Hg mines in the Lena district, whereas samples 14 and 15 were analogously collected downstream of the Mieres Hg mining district. Sample 12 is located downstream of a closed copper (Cu) mine. Sample 18 was taken in the Nalón river, after receiving the waters from the Caudal River and, therefore, outside the Caudal River basin. Four closely located sediment subsamples were mixed to obtain a homogeneous and representative sample at each point. The water was sampled in 10 points where sediment samples were also taken (numbers in blue color in Fig. 1). All the samples were collected on the banks of the rivers using plastic tools and containers and correctly labeled and preserved according to the analytical method to which they were to be subjected. Water parameters, such as pH or electrical conductivity, were measured in situ by means of a YSI multiparameter probe.

### Laboratory

At the laboratory, sediment samples were dried at room temperature (25 ± 5 °C) up to constant weight (1–2 weeks) to avoid Hg volatilization, and sieved so the fraction < 125 µm was selected for analysis, which was undertaken by ALS Laboratory Group. A 0.5-g sample was digested in the aqua regia acid mixture of nitric and hydrochloric acids in a 1:3 ratio at room temperature to maintain volatiles such as Hg, and analyzed using a combination of ICP-AES and ICP-MS determinations on the same sample to quantify 53 elements. The detection limit of this multi-element super trace analysis is 0.01 mg kg^−1^ for As and 0.004 mg kg^−1^ for Hg. In order to determine the mineralogy, the samples were subjected to X-ray diffraction (XRD) analyses by means of a Bruker D8 Discovery diffractometer and studied by scanning electron microscopy-energy dispersive X-ray spectrometry (SEM–EDS) using a JEOL 6610 LV unit, both available in the University of Oviedo facilities.

Water samples were filtered in situ and preserved with ultra-pure nitric acid (1 mL/145 mL) and 50 mL were analyzed directly by ICP-MS and ICP-AES in the same laboratory, and 53 elements were quantified in the samples. The detection limit is 0.05 μL^−1^ for both As and Hg. Anions (Br, Cl, F, nitrate, and sulfate) were analyzed by ion chromatography.

QC/QA included the analysis of sample duplicates (3% of the total number of samples), blanks, and certified reference materials (OREAS 45f, OREAS 46, OREAS 905, GBM908-10, and MRGeo08 for sediments and NIST 1643f, SM1 494–002, and WaterSTD-2 for waters) with recovery ratios between 95 and 100%.

### Biological analysis

The Hydrological Planning Office (Cantabrian Hydrographic Confederation) routinely evaluates the ecological state of the water bodies. Macroinvertebrates are one of the most widely used biological groups as indicators of water quality due to their great diversity and different taxa (Kenney et al. 2009), as well as the fact that they have different ecological requirements related to the hydromorphological, physicochemical, and biological characteristics of the aquatic environment (Menezes et al. 2010; CHEbro 2023). In June, 2022, a semiquantitative multihabitat sampling of macroinvertebrates was undertaken in three points of the Caudal River basin, using a 500-micron mesh size kick-net with a 25-cm frame width. Collected organisms were identified and organized into separate families. The sampling was originally performed to produce estimates of IBMWP (Iberian Biological Monitoring Working Party) index (Munné and Prat 2009), but we used raw abundance data of benthic invertebrates to calculate basic biodiversity indices: species richness (number of families) and dominance. Dominance was estimated using the Berger-Parker index, which is the relative abundance of the most abundant group: the higher the value, the lower the evenness and therefore the lower the biodiversity (Magurran 2004).

## Results and discussion

### Waters

Previous work undertaken by the Hydrological Planning Office in this basin for more than a decade shows that the waters in the head of the basin have a relatively high iron (Fe) content, due to a natural origin. Some high values ​​of ammonium, nitrates, or phosphorus, typical indicators of urban pollution or livestock activity, have been found in some points. Punctual elevated concentrations in metals or sulfates have been found in the low section of the basin. Notwithstanding, the watercourses located downstream of the Hg mines have not been regularly sampled by this public office.

Stream water sampled below mines might exceed the concentrations that the US Environmental Protection Agency indicates and this fact may result in chronic effects on aquatic life (USGS 2016). For example, concentrations up to 13 μg L^−1^ Hg and 0.03 μg L^1^ methyl-Hg were found in the Almadén Hg mining district in Spain (Gray et al. 2004), whereas concentrations of 100–5000 μg L^−1^ As have been reported in ground and surface waters near sulfide mineralization areas and smelting sites (Rahman et al. 2012).

Table 1 shows the concentrations of some metals and As found in water samples in this work. The criterion continuous concentration (CCC) for an element is an estimate of the highest total concentration of that element in freshwater to which an aquatic community can be exposed indefinitely without resulting in an unacceptable effect, i.e., it is not expected to pose a significant risk to the majority of species in that environment (criteria for metals are expressed in terms of the dissolved metal in the water column) (US EPA 2022a). The CCC values are shown in Table 1. The US EPA hardness-adjustment equation was used to calculate the US EPA’s CCC water quality criteria for Cu: CCC = 0.96 · *e*^0.8545^ ^· ln(*H*) −1.702^, where *H* is hardness expressed as mg L^−1^ as CaCO_3_ (US EPA 2022a; Meyer and Adams 2010). In this case, the hardness of the samples varies from 79 (slightly hard) to 405 mg L^−1^ CaCO_3_ (very hard). The lowest (most conservative) value was selected as CCC for Cu (8.3 µg L^−1^ Cu; Table 1). The Spanish regulation (RD 817/2015) establishes the Environmental Quality Guidelines (NCA) for water, defined as the “concentration of a certain pollutant in water which must not be exceeded for the protection of human health and the environment.” The threshold values for the metal(oid)s considered in this study are included in Table 1. For those elements whose limit is defined as dependent on water hardness, the most conservative values were selected, corresponding to the range from 50 to 100 mg L^−1^ CaCO_3_.

**Table 1:** Metal(oid)s concentrations in water samples (µg L^−1^) compared to guidelines

	Min	Max	Avg	NCA	CCC
As	0.09	762	177	50	150
Cd	< 0.005	0.013	0.006	0.09	0.72
Co	0.015	0.147	0.037	N/A	N/A
Cr	< 0.5	< 0.5	< 0.5	50	74
Cu	0.30	2.90	0.95	40	8.3
Fe	< 3.0	22.0	8.00	N/A	1,000
Hg	< 0.05	< 0.05	< 0.05	0.05	0.77
Mn	0.46	12.5	2.07	N/A	N/A
Pb	< 0.05	0.24	0.10	7.2	2.5
Sb	0.06	1.24	0.42	N/A	N/A
Se	< 0.05	0.23	0.12	1	N/A
Zn	0.50	5.50	1.99	300	120

*N/A*, not available.

The CCC for As is widely exceeded in the watercourses downstream of the Hg mines in the Lena (samples 9 and 10) and Mieres (sample 15) mining districts. The As concentrations in waters sampled at these three points are also high above the NCA (up to 15 times). However, since Hg is less soluble, Hg concentrations in these surface waters are below the NCA, the CCC, and the 1.0 μg L^−1^ Spanish drinking water standard. Although the concentrations of the rest of metals are very low and all are below the considered threshold levels (Table 1), it can be noted that the highest values of Cu, Pb, and Zn were found in sample 11, taken downstream of some metal processing industries near the main tributary of the Caudal River (Fig. 1). Other relatively high values of these metals are reached downstream of the Hg mines of Mieres and Lena districts, since the existence of sulfides of these metals is documented in the paragenesis of the Hg deposits, as it stated in the “Area of study” section.

**Fig. 2:**
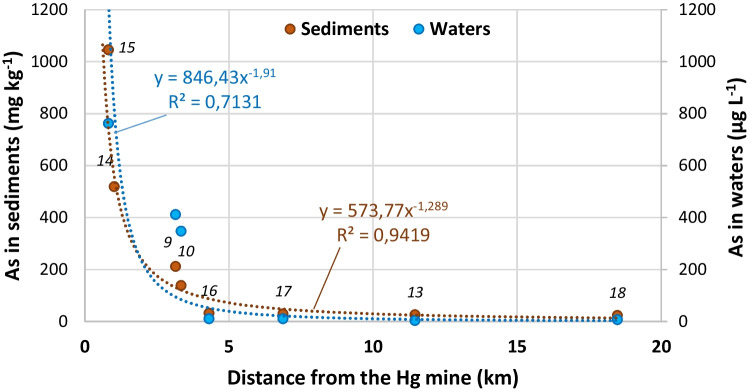
Decrease of As concentrations in sediment and water samples as a function of distance from the Hg mines

It is essential to define the drainage basins, especially those in which the mines are located, in order to select the sampling points that best characterize the influence of these sources on the aquatic environment. Depending on the order of the watercourses, the water flow of those sampled varied from 0.1 (point 15, brook downstream of a Hg mine, tributary of the Caudal River) to 27 m^3^ s^−1^ (point 17, final section of the Caudal River). All the sampled waters are neutral (pH 7 to 8), with light or moderate hardness, and with very weak to weak mineralization, with the exception of the watercourse downstream of the main Hg Mieres mine (sample 15), whose waters are very hard and of moderate mineralization (electrical conductivity above 0.7 mS cm^−1^). This last sample is calcium sulfate, while all the rest are calcium bicarbonate. The concentration of As is high in the streams that drain the mining areas, but when they flow into the Caudal River, the water As content is much lower, due to the dilution effect of a greater flow. Figure 1 shows how the Hg and the As contents decrease considerably with increasing distance to the mines and the Strahler stream order. This is specifically shown in Fig. 2, where the concentrations of As in waters and sediments are represented against the distance from the source (Hg mines). Samples 9, 10, and 13 are related to mines in the Lena district and the rest to mines in the Mieres district. Sample 18 was taken in the Nalón river, outside the Caudal River basin. A potential fit has been performed and, although the fit is better for sediments (*R*^2^ = 0.94) than for water (*R*^2^ = 0.71), a rapid decrease in concentrations with distance from the source is observed, so that from about 4 km onward baseline values are reached. However, the average mass load of As entering the River Caudal from the main Hg mines has been estimated in about 10 tonnes per year (Ordóñez et al. 2014). Part of this is As that is retained in the sediments, as will be seen in the next section.

### Sediments

**Fig. 3:**
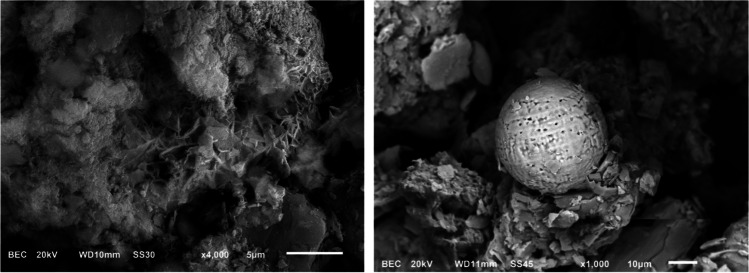
SEM images obtained in sediment samples: a mixed particle containing As-rich Fe oxides (left) and a spherical particle of fly ash (right)

XRD has made it possible to identify the majority crystalline phases of the samples, which are a reflection of the mineralogical constitution of the substrate rocks, being quite homogeneous and dominated by silicates. Quartz and Al–K silicates (almost always in the form of muscovite and its weathering products) are the two dominant species and usually constitute more than 95% of the identified species. Chlorite-group minerals, which are associated with shale substrates, are identified in some samples. Carbonates (calcite and dolomite) reach 3% in samples from the lower half of the basin,. The As phase most commonly associated with Fe is that which adsorbs to Fe oxides or oxyhydroxides, namely, amorphous Fe (Ollson et al. 2016). SEM–EDS analysis has allowed to find, in samples taken downstream of the Hg mines, particles of clays and Fe oxides of high specific surface area, enriched in As. This suggest a mechanism of chemical dispersion: the As is released from the As minerals in the mining areas (realgar, orpiment, arsenopyrite, or As-rich pyrite) into the aquatic environment; in the sediments, it appears associated with Fe oxides but it does not form its own minerals, so the As is leached, transported in solution, and retained onto mineral surfaces of the sediments. Figure 3 shows a particle with needle-shaped precipitates of Fe oxides containing As; this shape indicates they were formed by means of a surficial process. This figure also shows a spherical and porous particle of fly ash, found in fluvial sediments sampled downstream of a thermal power plant.

Sediments act as sinks, as they have the capacity to accumulate and integrate over time elements present in the water, even if their concentrations are extremely low and undetectable or even long after the original sources of contamination have been removed (Soares et al. 1999).

Table 2 shows Hg and As concentrations in sediments in some mining areas around the world. Sediment samples collected from streams in unmineralized areas usually contain less than 1 mg kg^−1^ Hg, but those collected close to Hg mines can reach much higher concentrations (USGS 2016). Almadén was the largest Hg mining district in the world (García-Sánchez et al. 2009). Hg content in stream sediments reached up to 2300 and 4100 mg kg^−1^ Hg in Almadén and Idrija Hg mining districts (probably the most polluted sites of this type in the world), respectively, and those collected near the Almadén mine contain up to 82 mg kg^−1^ methyl-Hg (Gray et al. 2004; Gosar 2008). In other studies, sediment As concentrations ranged from 6 to 25,000 mg kg^−1^, being the latter in polluted areas (US EPA 2022a; WHO 2001; Correa et al. 2021; Rieuwerts et al. 2014). In most cases, the highest values correspond to sediments taken just downstream of the mining sites, whereby tailing piles or the river bed itself are eroded by the rivers, so that fine-grained fragments of calcine or cinnabar particles are washed away (Gray et al. 2004; Gosar 2008).

**Table 2:** Hg and As concentrations in sediment samples found in mining-influenced basins

	Hg (mg kg^−1^)			As (mg kg^−1^)			
River basin	Min	Max	Avg	Min	Max	Avg	Ref
Guadiana (Spain)	0.16	4.43	0.86	8.8	81.8	29.5	Delgado et al. (2011)
Tamar (UK)				800	25,000	9400	Rieuwerts et al. (2014)
Valdeazogues, Almadén (Spain)	1.77	175	106	7.15	16.0	12.7	García-Ordiales et al. (2014)
Azogado, Almadén (Spain)	770	2300	1400				Gray et al. (2004)
Idrijca, Idrija (Slovenia)	32	4121	734				Gosar (2008)
Río Tinto (Spain)				250	3,090	864	Galán et al. (2002)
Upper Isle (France)	0.04	0.25	0.12	159	889	418	Grosbois et al. (2007)
San Francisco, Apocho (Chile)	0.005	0.35	0.05	6.0	25.6	18.7	Correa et al. 2021

Table 3 shows the range and mean concentrations of the main elements found in the studied sediment samples, as well as their relative dispersion. In terms of Hg and As, the concentrations of the sediments from the Caudal River basin are in the range of those of other basins affected by mining (Table 2); the sediments downstream of the mines are clearly contaminated but not as much as other mining districts such as Almadén or Idrija. The coefficient of variation or relative dispersion is elevated for Hg and As in the sampled sediments, indicating their heterogeneous distribution in the basin, since the background samples have low concentrations, whereas the maximum values are found in the vicinity of the Hg deposits of the Lena and, particularly, the Mieres districts (Fig. 1). Other elements, such as Fe or Mn, have a low relative dispersion, corresponding to a homogeneous distribution due to their natural or geogenic origin.

**Table 3:** Descriptive statistics of metal(oid)s concentrations in sediment samples (mg kg^−1^) and background levels, compared to guidelines

	Min	Max	Avg	Rel. disp.	Bgnd	TEL	PEL	FSSB
As	9.31	1,045	126	2.14	13.3	5.9	17	9.8
Ca	1700	65,400	15,365	1.01	9600	N/A	N/A	N/A
Cd	0.08	0.47	0.14	0.65	0.13	0.6	3.5	0.99
Co	8.07	34.5	12.6	0.48	12.6	N/A	N/A	50
Cr	8.51	24.2	13.3	0.31	17.2	37.3	90	43.4
Cu	12.9	51.9	20.7	0.46	19.3	35.7	197	31.6
Fe	23,400	36,600	28,500	0.13	30,700	N/A	N/A	20,000
Hg	0.05	195	18.6	2.65	0.08	0.17	0.486	0.18
Mn	350	1460	519	0.25	499	N/A	N/A	460
K	400	1,000	559	0.49	640	N/A	N/A	N/A
Ni	18.8	61.4	28.3	0.37	31.9	N/A	N/A	22.7
Pb	14.0	34.7	19.7	0.30	19.7	35	91.3	35.8
Sb	0.45	1.68	0.78	0.47	0.78	N/A	N/A	2
Se	0.14	0.56	0.29	0.36	0.31	N/A	N/A	2
Zn	52.6	332	84.7	0.76	73.0	123	315	121

*N/A*, not available.

Table 4 shows the Pearson’s correlation coefficient matrix of the studied elements, obtained by means of the software IBM SPSS Statistics vs 27. The concentrations of Hg and As are highly intercorrelated (*r* = 0.91), whereas the correlation between other metals is not remarkable, with the exception of the elements Zn–Cd–Co–Mn, which are quite correlated with each other. The Zn–Cd relationship is a well-known natural elemental association linked to the existence of sphalerite in the mineral paragenesis of Hg deposits (mentioned above). Mn oxides are low-temperature crustiform minerals, often associated with their analogous Co oxides and oxyhydroxides (such as heterogenite). On the other hand, Cr and Fe concentrations seem quite correlated, being two elements with very similar chemical properties. Given the low concentration of Cr in the sediments and the inexistence of specific minerals of this metal in the studied basin, the most probable hypothesis is that Cr appears substituting Fe in the species that include it. In any case, it should be noted that the concentrations of Zn, Cd, Co, and Cr are low in the sediments studied.

**Table 4:** Pearson’s correlation coefficient matrix (*n* = 17; *significant at 0.05 level (two tailed); **significant at 0.01 level; the highest coefficients are indicated in bold)

	As	Ca	Cd	Co	Cr	Cu	Fe	Hg	K	Mn	Ni	Pb	Sb	Se	Zn
As	1														
Ca	0.177	1													
Cd	0.376	0.742^**^	1												
Co	0.363	0.669^**^	0.963^**^	1											
Cr	− 0.326	− 0.077	0.266	0.254	1										
Cu	0.156	0.283	0.459	0.528^*^	0.195	1									
Fe	0.125	0.053	0.468	0.440	0.815^**^	0.228	1								
Hg	0.907^**^	− 0.242	− 0.048	− 0.047	− 0.401	− 0.014	− 0.046	1							
K	0.241	0.165	0.106	0.170	0.092	0.234	0.046	0.188	1						
Mn	0.464	0.743^**^	0.966^**^	0.976^**^	0.178	0.463	0.407	0.039	0.251	1					
Ni	0.160	0.532^*^	0.912^**^	0.921^**^	0.592^*^	0.456	0.693^**^	− 0.220	0.128	0.873^**^	1				
Pb	0.033	0.247	0.292	0.167	0.408	0.035	0.519^*^	− 0.066	− 0.175	0.199	0.296	1			
Sb	0.683^**^	0.158	0.443	0.316	0.161	0.101	0.509^*^	0.518^*^	0.094	0.381	0.355	0.466	1		
Se	0.506^*^	0.542^*^	0.746^**^	0.702^**^	0.308	0.314	0.626^**^	0.206	0.314	0.723^**^	0.700^**^	0.472	0.656^**^	1	
Zn	0.328	0.804^**^	0.970^**^	0.953^**^	0.152	0.358	0.369	− 0.119	0.038	0.955^**^	0.864^**^	0.251	0.341	0.704^**^	1

The background values found in this study (Table 3) for Hg and As are 0.08 and 13.3 mg kg^−1^, respectively (arithmetic mean of concentrations in samples 1 to 5, taken in the highest part of the basin). The contents of these two elements in the rest of the samples are similar to the background value, except for those taken downstream of the Hg mines. Samples taken downstream of Mieres Hg mining district (e.g., #15, containing 195 and 1045 mg kg^−1^ of Hg and As, respectively) exceed more than 2000 and 70 times these background values of Hg and As, respectively, which this gives an idea of the magnitude of the pollution caused by former Hg mining activities. Samples taken downstream of Lena Hg mining district (#9 and 10) exceed 70 and 200 mg kg^−1^ of Hg and As, respectively, whereas the concentrations of Hg and As in the Caudal River, downstream of both districts (samples #16 and 17) are around 1 and 30 mg kg^−1^, respectively. Thus, all the samples taken downstream of the Hg mines are clearly above the background levels for both elements. As for the rest of the metals, all the samples have concentrations similar to the respective calculated background values. Only sample 12, which exceeds the background value for Cu by 2.7 times, and sample 14, which exceeds the background values for Zn and Cd by 4.5 and 3.6 times, respectively, stand out. These samples are commented below. The concentrations of elements such as Co, Pb, Mn, Sb, Ni, and Se, typical from hydrothermal deposits, are slightly above their background in samples taken downstream of the main Hg mine sites. Among the elements of natural origin, the Ca content in the sampled sediments agrees with the distribution of carbonate and/or marly rocks in the basin, since it is higher than its background in samples 2, 12, 13, 14, 16, and 17, i.e., it is predominant in the lower part of the basin. The high Ca content may be favorable, as Rodríguez-Martín et al. (2009) found that the calcareous nature of the mineral particles (soils in that work) minimizes the negative effect of Hg load; Hg immobilization is favored by high pH and organic matter and clay contents (Rodríguez-Martín et al. 2013b). The K content is also related to the existence of shaly rocks, and its maximum is reached in sample 2.

**Fig. 4:**
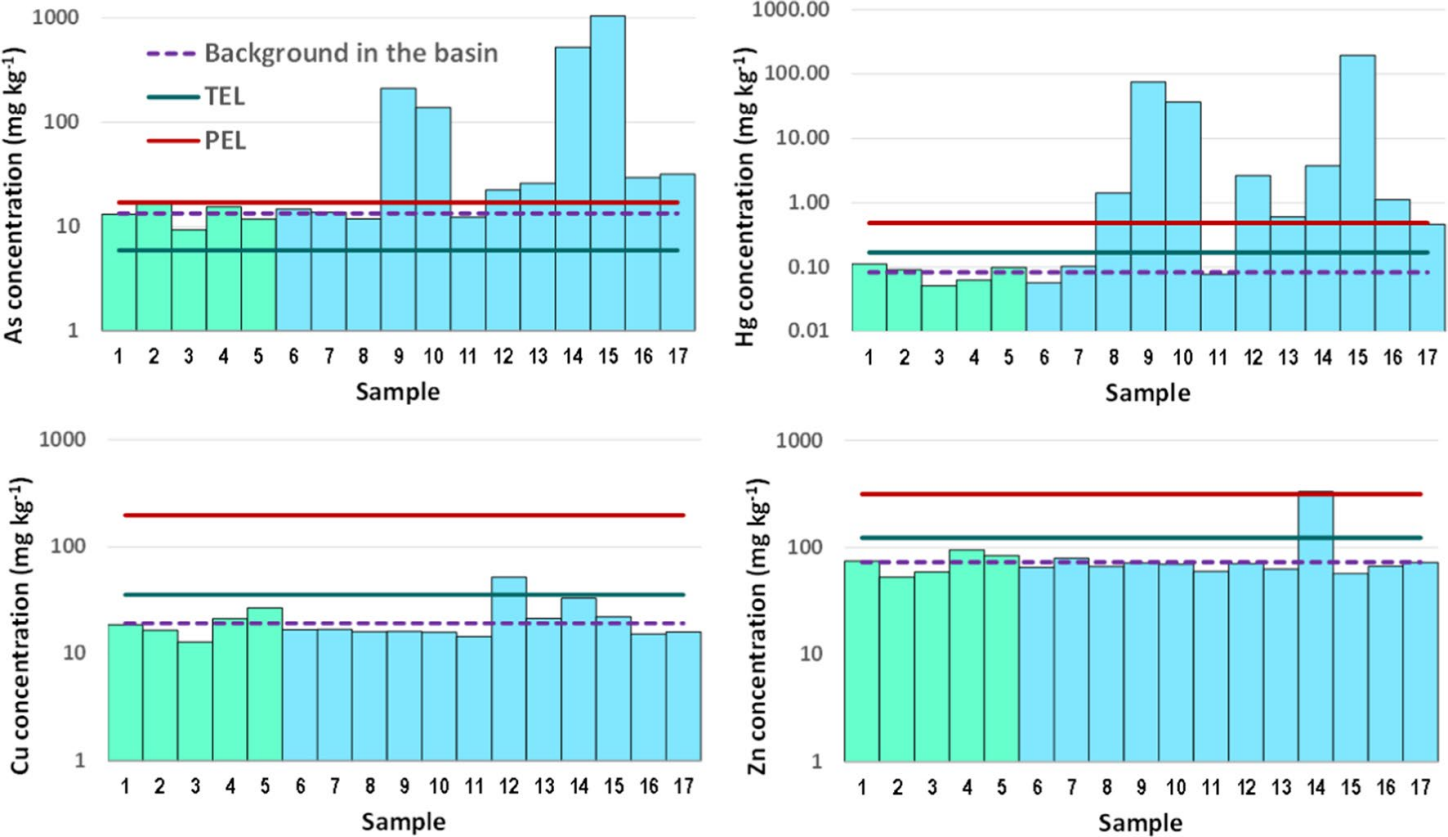
Concentrations of As and Hg in the sediment samples compared to the background and threshold levels. Samples in green are those selected for background. Note logarithmic scale

In contemporary aquatic ecosystem protection and management, the evaluation of sediment-associated metals’ toxicological significance has emerged (Caballero-Gallardo et al. 2020). Comparison of contaminant concentrations with sediment quality guidelines (SQG) is a common approach to assessing sediment quality by establishing a relationship between the expected incidence of toxicity and the exceeded guideline value, and is therefore used to prioritize contaminated areas for further investigation or to establish spatial patterns, although it has some limitations due to uncertainties associated with site-specific characteristics (Violintzis et al. 2009). Table 3 compares the concentrations of As and the main metals found in sediment samples to some commonly used and accepted threshold levels. The Canadian Sediment Quality Guidelines for the Protection of Aquatic Life set threshold effect levels (TELs) and probable effect levels (PELs) for several pollutants, in order to evaluate the degree to which adverse biological effects are likely to occur as a result of exposure to those elements in sediments (CCME 2002). In this study, As, Hg, Cu, and Zn are above these limits. All the samples exceed the TEL for As, due to a naturally high geochemical background related to the mineral deposits and 53% of the samples are above the PEL, the probable effect range within which adverse effects frequently occur. The content of Hg in 47% of the samples is below the TEL (minimal effect range within which adverse effects rarely occur), but in 47% of them the PEL is exceeded and 6% is between both thresholds (the possible effect range within which adverse effects occasionally occur). For both elements, the PEL is exceeded by more than an order of magnitude in the sediments downstream of the Hg mines, so negative effects on aquatic life should be expected. Figure 4 compares the As, Hg, Cu, and Zn contents in the sediment samples to the calculated background levels and the PEL and TEL threshold values. Ancient mining activities are also considered to be the responsible for the high Cu (sample 12) and Zn (sample 14) contents. The highest Cu concentration was found in sample 12, which was taken in a stream that drains the basin where an old Cu mine is located. In this site, several thousand cubic meters of malachite/azurite-rich mine wastes and slags are disposed over steep slopes. Regarding sampling 14, it is located in a waterbody that drains the basin in which the Los Rueldos Hg mine is located (Mieres district). According to Loredo et al. (2005), metallic mineralization at Los Rueldos mine site includes, in addition to Hg and As species, sphalerite (secondary smithsonite and hemimorphite) and chalcopyrite (secondary malachite), which explains the relatively high Zn content; the usual presence of Cd as impurity in sphalerite can explain the relatively high concentrations of this metal in sample 14.

In addition, most samples exceed the US Environmental Protection Agency Freshwater Sediment Screening Benchmark (FSSB, Table 3) (US EPA 2022b) for As and half of them exceed this threshold for Hg (Table 3). Although the PEL and TEL levels are not defined for Co, Fe, Mn, Sb, and Se, there are FSSB values for these elements. The concentrations of the sampled sediments are below these values, except for Fe and Mn. These are common elements in the rocks that constitute the Earth crust and their respective clarkes are 36,500 mg kg^−1^ Fe and 774 mg kg^−1^ Mn (Moon et al. 2006). Álvarez et al. (2010) found contents of 45,600 mg kg^−1^ Fe and 670 mg kg^−1^ Mn in the lutites of the Fresnedo Group, located in the upper part of the Caudal River basin. Fe-bearing minerals are phyllosilicates, mainly clinochlore, whereas the Mn appears in the form of different oxides. This being the case, a natural origin for these two elements has to be proposed, being remarkable the relatively low value of the FSSB for both elements. Co, Sb, and Se concentrations are in all cases below their respective FSSB standards.

**Fig. 5:**
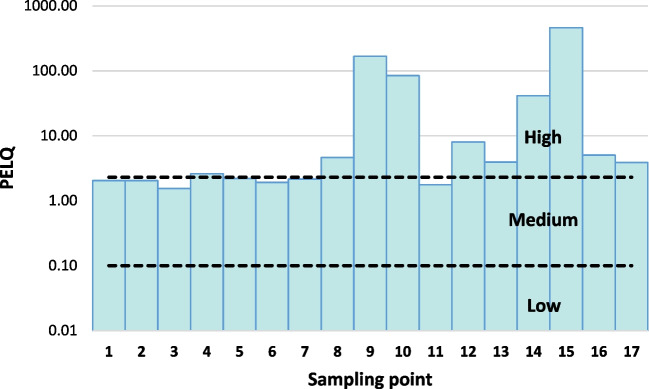
Mean PEL quotient for each sampling point and priority in terms of toxicity

A more realistic measure of predicted toxicity, the average PEL quotient (PELQ) was calculated according to this equation (Violintzis et al. 2009):

$$PELQ= \frac{\sum \left[\frac{{C}_{i}}{{PEL}_{i}}\right]}{n}$$

where *C*_*i*_ is the concentration of element *i* in the sediment, PEL_i_ the guide value for element *I*, and *n* is the number of metals considered. This average quotient is useful for reducing many pollutants to a single number. Adverse effects to aquatic organisms caused by individual chemicals are assumed to be additive, but not all chemicals present in the sediment are considered, only those included in the guideline list. PELQ values of < 0.1, 0.1–2.3, and > 2.3 coincide with 10%, 50%, and 76% likelihood of toxicity, respectively, which define four relative levels of priority: high, medium, and low (Long and MacDonald 1998, Violintzis et al. 2009). Figure 5 shows the mean PELQs calculated for the sampling points, based on the metals As, Cd, Cr, Cu, Hg, Pb, and Zn. These sampling points are distributed between the medium and high priority classes. In particular, sediments from points 9, 10, 14, and 15 (those taken downstream of Hg mining sites) showed the highest proportions, mainly due to their high Hg content in relation to the PEL, and might present a significant risk to biota.

Nevertheless, a rapid reduction of concentration with the distance from the source has been found (Figs. 1 and 2). As we move downstream of the Mieres Hg mines in the Caudal River toward the Nalón River, the As concentration in the sediments decreases drastically (about 40 times in 4 km). Despite this, it cannot be ignored that at a distance of 50 km downstream of the source (Hg mines), the As concentrations in sediments still exceed the PEL, posing a threat to the aquatic life. Results suggest a definite probability of adverse effects to the local aquatic biota in the lower part of the basin. However, according to the historical data obtained by the Hydrological Planning Office at the sampling point equivalent to point 15 of this study, there has been a decrease in the As content since 2016 in this area.

### Biological medium

The integration of biological parameters in addition to physico-chemical measurements has proven to be a more comprehensive method for fully assessing the effects of contaminants in aquatic ecosystems, since bioassessment offers greater reliability in assessing the presence and impact of pollutants, particularly by means of the use of macroinvertebrates as bioindicators. Macroinvertebrates are sensitive to aquatic contaminants so they can constitute reliable indicators of the presence of metals in lotic systems due to their intimate contact with the sediments, their relative immobility, and their prolonged residence time. They also represent the main source of food for many organisms and therefore play an important role in the transmission of pollutants to higher levels of the trophic chain (Eyong 2008; Hare 1992; Karr 1991; US EPA 1988). Although metal uptake is variable depending on organisms, positive correlations between metals in sediments and those in macroinvertebrates have been found in numerous studies and cases of bioaccumulation and biomagnification of metals have been reported (Eyong 2008; Farag et al. 2007). Also, macroinvertebrates taxa and individuals tend to reduce in number in rivers polluted from metal mining (Roline 1988). Culioli et al. (2009) found that 70 years after mining ceased, As concentrations in water, sediments, and biota were high in the Bravone River basin (Corsica).

In this study, although there are high concentrations of some metals at some points, the driving element is As, both because of its toxicity and its dispersion in the basin, followed by Hg. As is biologically available to aquatic organisms living in contaminated environments; it can be found in higher concentrations in macroinvertebrates than in the water, although its accumulation differs among taxa according to their feeding habits (e.g., it is higher in shredders than in predators), indicating the relevance of detrital processes in As accumulation (Culioli et al. 2009). Regarding Hg, Sinha et al. (2007) found bioaccumulation factors up to 500 in macroinvertebrates exposed to Hg pollution. Once Hg enters the aquatic environment, even at low concentrations, biomagnification processes can raise the concentration of organisms to toxic levels (Han et al. 2023).

A surface water body is considered to achieve a “good” ecological state if the values of the indicators of the biological quality show low values of distortion caused by human activity, slightly deviating from the values normally associated with the type of water body under undisturbed conditions. According to the Hydrological Planning Office, the ecological state of the watercourses in the lower basin of the Caudal River is not good in some sections due to the existence of sources of diffuse contamination (agricultural and livestock activity) and specially punctual sources (industrial discharges and mainly mining activity). In fact, the ecological state is considered poor or “deficient” in the watercourses downstream of the closed mines. In this case, the desired values of the biological indicators of aquatic macroinvertebrates are not reached since the chemical indicators do not comply with the ranges that guarantee the functioning of this type of ecosystem, given that the concentrations of specific contaminants such as As are above the NCA. This has been proven by means of a canonical correlation analysis, which was performed between the sets “biodiversity” (richness, dominance) and “contamination” (Hg, As, Zn) by means of the software IBM SPSS Statistics vs 27. Cut-off correlations of *R*_C_ > 3.0 were used for interpretation of the canonical variates (Parrington et al. 2013; Balkin et al. 2011; Tabachnick and Fidell 2007). A statistically significant relationship was found between both sets. The first canonical function was significant (*p* = 0.002), *F* (6, 12) = 6.84, eigenvalue = 16.3, and explained 95% of variance shared between the variable sets. This effect was calculated from the Wilks test value (*λ*) by *R*_c_^2^ = 1 – *λ* (i.e., 1 − 0.051 = 0.949). The second canonical root was not significant, so only the first canonical variate was interpreted. In order to assess the direction and contribution of the variables, the structure coefficients were assessed. Table 5 shows the correlations and standardized canonical variate coefficients for “contamination” and “biodiversity” subscales, as they relate to the first canonical variate. This variate included positive scores on the subscales of the “contamination”: Hg, As, and Zn (only high for the first two). The canonical loadings are high for both richness and dominance, but negative for richness. This means that when Hg and As concentrations are elevated in the sediments, richness clearly decreases, whereas dominance increases, so biodiversity is reduced and so does the goodness of the ecological state of the river.

**Table 5:** Correlations to the canonical variate and standardized canonical variate coefficients for the first canonical variate

	Correlation to the canonical variate	Standardized canonical variate coefficient
Contamination scale		
Hg	0.952	0.818
As	0.950	− 0.017
Zn	0.695	0.341
Biodiversity scale		
Richness	− 0.995	− 0.865
Dominance	0.854	0.162

**Fig. 6:**
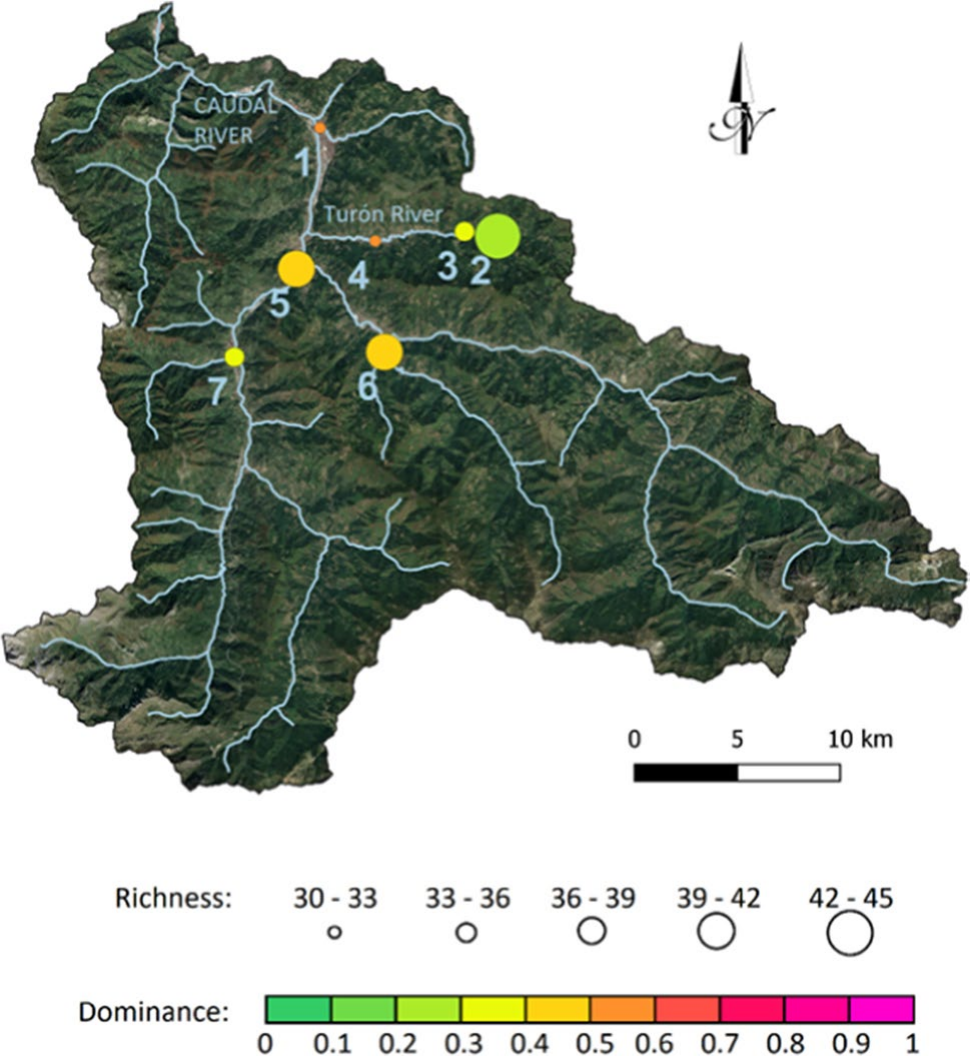
Average richness and dominance found for the sampled macroinvertebrates in the Caudal River basin in 2018–2022

The biodiversity study in this case shows that the most abundant macroinvertebrate families in the sampled points are Chironomidae, Baetidae, Acariformes, and Hydrobiidae. Exposure of communities to heavy metals often has effects such as reduced abundance, decreased species richness, and altered community composition, mostly consisting in biodiversity loss. In this case, even though there is a wide dispersion, a negative correlation between richness and dominance has been found, since when a greater number of species coexist, the community is usually not so dominated by a single group. Figure 6 shows the average values of both parameters in the last five campaigns (2018–2022) at the points sampled in the Caudal River basin. Expressing the results in the form of averages allows for the simplification of information, highlights general trends, and facilitates comparison across different matrices. Family richness is lower downstream of the closed mines (points 1 and 4) and moderate in the rest of the points, while the dominance is also high in those points, suggesting a worse state of the ecosystem. Richness in point 7 is relatively low, likely because it corresponds to a stream that drains a basin where a small closed Hg mine of the Lena district is located. Point 1 is located downstream of one of the most important Hg mines of the Mieres district. In addition, it can be seen that biodiversity is reduced along the Turón river (points 2–3-4), which drains a sub-basin with several closed coal mines.

Table 6 compares the macroinvertebrate families found in summer 2022 in point 7 and point 1 (Fig. 6). Since point 1 is downstream of the old Hg Mieres mining district and it is the most affected of those sampled. Regarding the richness, only 15 families were found in this point, whereas 28 were identified in point 7. In contrast, a few species, such as Hydrobiidae—followed by Acariformes and Chironomidae by far—clearly predominate over the rest in point 1, so that the dominance at this point is more than twice as high as at point 7. Hydrobiidae was found by Marqués et al. (2003) as one of the most tolerant families to pollution from Zn–Pb mining in a fluvial ecosystem in Spain. Chi et al. (2017) found that the chironomidae species occupied a dominant position showing its high tolerance to high concentrations of As in a Chinese river, so its high presence in point 1 is not surprising. Notwithstanding, in general, taxa such as Chrironomidae are extensively adaptable to the environment and occur in almost all water quality grades (Xu et al. 2014), so the amount of specimens of this family found in point 7 is one order of magnitude higher than that of point 1. On the other hand, the Acariformes are considered to be a relatively tolerant family (Dunlop et al. 2008), hence their dominance in point 1.

**Table 6:** Number of specimens of macroinvertebrate families found in points 1 and 7 (Fig. 6) in June, 2022, and calculated values of richness (in bold) and dominance (in bold)

Family	Point 7 (Fig. 6)	Point 1 (Fig. 6)
Acariformes	160	608
Baetidae	1616	1
Chironomidae	2226	288
Dryopidae	1	16
Elmidae	96	96
Empididae	96	16
Ephemerellidae	704	
Gerridae	1	
Haliplidae	16	
Heptageniidae	1	
Hydrobiidae	144	4864
Hydrophilidae	1	
Hydropsychidae	16	1
Hydroptilidae	16	
Leptoceridae	1	1
Leuctridae	272	32
Limnephilidae	1	
Lymnaeidae	128	18
Nepidae	1	
Oligochaeta	64	1
Polycentropodidae	48	
Psychodidae	16	
Physidae		32
Rhagionidae	1	
Rhyacophilidae	1	1
Sericostomatidae	1	
Simuliidae	368	16
Sphaeriidae	48	
Tipulidae	1	
Total	6045	5991
**Richness**	**28**	**15**
**Dominance**	**0.37**	**0.81**

## Conclusions

i. Despite significant improvements in the relationship between mining activities and the environment in recent decades, some old mining sites continue to pose a risk to ecosystems. This is the case of the Caudal River basin, where these effects are noticeable in the quality of the water and river sediments. The concentrations of Hg and As are naturally high in these media, as it is shown in the geochemical background levels found for the sediments. The analysis of the water and sediment samples allows to prove that the legacy of the old mining activities carried out in times when environmental regulations were less demanding than today continues to leave its mark, since the concentrations of Hg and As, and to a lesser extent, of Cu and other metals are high downstream of the former Hg and Cu mining sites, significantly exceeding background levels (up to 2000 times in the case of Hg).
ii. The mineralogical study of the sediment samples, combined with EDS microanalysis, is useful to identify geochemical traps such as Fe oxides or clays to retain the As that travels dissolved from the sources. In a large watercourse like the Caudal River, high concentrations of As are not detected in the water, but the retention of this toxic metalloid in the sediments can be very harmful to aquatic ecosystems. In fact, the elevated Hg and As (and to a lesser extent, Cu and Zn) concentrations that have been found downstream of the old mines were above the considered SQG, suggesting that adverse biological effects may occasionally or frequently occur. The mean PELQ values, calculated from the concentrations of As, Cd, Cr, Cu, Hg, Pb, and Zn in the sediments, show that the sampled area is in the medium–high priority in terms of probability of toxicity, mainly due to the high Hg content, so it could present a significant risk to biota.
iii. Traditional physicochemical analytical techniques do not provide a full assessment of the impact of the contaminants on the overall health of the aquatic ecosystem so a bioassessment is required, being macroinvertebrate-reliable biomonitors of metal pollution in lotic systems. The study of macroinvertebrates shows that richness is low and dominance is high right downstream of these sources, since the number of specimens is comparatively low and only a few tolerant and adaptable species, such as Hydrobiidae, clearly dominate. A canonical correlation analysis has proven that when Hg and As concentrations are elevated in the sediments, richness clearly decreases whereas dominance increases, so the negative effects of Hg and As on aquatic life are reducing biodiversity, since there are signs of significant qualitative and quantitative restrictions of invertebrate fauna. The Hydrological Planning Office establishes that the Caudal River is not in a good ecological state, especially in its lower part, being considered to have a poor state in some sections and the results of this study have revealed the continuous and silent contribution from the mining sites as responsible for that. This is mainly due to punctual sources, such as old Hg mining sites that, despite being inactive for 5 decades, continue releasing contaminants whose high concentrations affect the biological indicators.
iv. This type of studies must be approached holistically, considering geological aspects (mineralogy of rocks and mineralizations that may release pollutants into the aquatic environment), hydrological aspects (layout and order of waterbodies and delimitation of drainage basins where pollution sources are located), and biological aspects (analysis of organisms that will be the best indicators of the degree of affection that these sources cause to the aquatic ecosystem).
v. It would be recommendable to continue studying the sediments of this basin to deepen the relationship of the geochemical data with the geological substrate, the anthropic influence (mining), and the aquatic organisms (macroinvertebrates and also small fish, such as piscivores). It would also be advisable to carry out water sampling throughout a hydrological year to evaluate the influence of meteorological conditions on the release of pollutants and the dilution of elemental concentrations in the water due to flow variations. Finally, it is necessary to definitively protect this aquatic environment from pollution by acting on the main sources (e.g., spoil heaps) to passivate them and stop them from releasing pollutants into the environment.

### Supplementary Information

Below is the link to the electronic supplementary material.

Supplementary file1 (XLSX 10 kb)

## Data Availability

Not applicable.
